# Ex vivo nanoscale abluminal mapping of putative cargo receptors at the blood-brain barrier of expanded brain capillaries

**DOI:** 10.1186/s12987-024-00585-x

**Published:** 2024-10-14

**Authors:** Mikkel Roland Holst, Mette Richner, Pernille Olsgaard Arenshøj, Parvez Alam, Kathrine Hyldig, Morten Schallburg Nielsen

**Affiliations:** 1 Department of Biomedicine, Aarhus University, Aarhus C, 8000 Denmark; 2 Laboratory of Neurological Infection and Immunity, Rocky Mountain Laboratories, National Institute of Allergy and Infectious Diseases, National Institutes of Health, Hamilton, MT 59840 USA; 3 Biotherapeutic Discovery, H. Lundbeck A/S, Valby, Copenhagen, 2500 Denmark

**Keywords:** Blood‒brain barrier, Transferrin receptor, Sortilin, Basigin, Porcine brain endothelial cells, Cargo receptor, Machine leaning and expansion microscopy.

## Abstract

Receptor mediated transport of therapeutic antibodies through the blood-brain barrier (BBB) give promise for drug delivery to alleviate brain diseases. We developed a low-cost method to obtain nanoscale localization data of putative cargo receptors. We combine existing ex vivo isolation methods with expansion microscopy (ExM) to analyze receptor localizations in brain microcapillaries. Using this approach, we show how to analyze receptor localizations in endothelial cells of brain microcapillaries in relation to the abluminal marker collagen IV. By choosing the thinnest capillaries, microcapillaries for analysis, we ensure the validity of collagen IV as an abluminal marker. With this tool, we confirm transferrin receptors as well as sortilin to be both luminally and abluminally localized. Furthermore, we identify basigin to be an abluminal receptor. Our methodology can be adapted to analyze different types of isolated brain capillaries and we anticipate that this approach will be very useful for the research community to gain new insight into cargo receptor trafficking in the slim brain endothelial cells to elucidate novel paths for future drug design.

**Supplementary Information:**

The online version contains supplementary material available at 10.1186/s12987-024-00585-x.

## Introduction

Bio-therapeutic antibodies show great promise for treatment of brain diseases, but this drug type faces the great challenge of having to enter the brain via the BBB. Brain endothelial cells (BECs) are gatekeepers between the blood supplying the brain and neuronal parenchyma that holds essential nutrients. They perform a unique function to both establish a barrier and mediate regulated transport of vital brain components due to expression of specialized regulators, which support this dual role [13]. The complexity of this transport reaches further than the BEC layer as a multitude of other cells both aid and perform to support BECs and the integrity of the capillary network to interface with the brain parenchyma [4]. The capillary network is built like a tree with main artery and vein trunks branching into penetrating vessels that branch further into the thinnest capillaries, which we here annotate microcapillaries. Microcapillaries, reach deep into the brain parenchyma where their average intercapillary distance is about 40 μm [5, 18]. Microcapillaries mainly consist of BECs with few pericytes supporting regulation of the vessel diameter [1].

Microcapillaries are of main interest for drug delivery because they are located within the shortest distance to many neighboring neurons thereby enabling drug delivery to the drug target site. The obstacle for reaching the target site is how the drug can pass through BECs when BECs themselves form a mechanical barrier. Receptor mediated transport (RMT) is a promising approach for delivery of therapeutics through BECs to the brain [15]. This approach relies on the availability of cargo receptors on the BEC luminal membrane surface so that these receptors can interact with the drug and convey its transport to neurons via RMT. Currently, little is known about intracellular machineries that control transcytosis in BECs, as techniques to study these mechanisms are limited [24].

However, recent technical advances have improved localization of biomarkers in the cells of microcapillaries, enforcing new strategies for drug delivery via BEC RMT: Single cell sequencing shows promise to better understand the cell zonation in deeper penetrating capillaries of the human brain [7]. This technology can potentially help to identify expression of novel cargo receptors for drug delivery through the microcapillaries. Further, transcriptomics combined with proteomics have allowed identification putative cargo receptors [28] and spatial proteomics has been applied to identify luminally positioned cargo receptors in isolated brain capillaries [10]. Whereas these methodologies enable crude identification of putative cargo receptors they are unable to report if putative cargo receptors are adequately localized to facilitate drug delivery via transcytosis. For this purpose, a technique is warranted that can report the nanoscale receptor localization within BECs in vivo. A such technique should reveal whether putative receptors are luminally, intracellularly and/or abluminally localized in BECs, ultimately enabling localization of therapeutic biologics as well as their transport fate through BECs.

This challenge may be approached by an imaging technique known as ExM by which nanoscale resolution of molecules in cells can be obtained [2]. The technique has proven useful for dissecting protein localization in complex tissues using a relatively simple and low-cost setup [23], and holds the potential to dissect localizations of putative cargo receptors in brain microcapillaries. For this purpose, we have in this study set up an ex vivo protocol to analyze receptor localization in microcapillaries of domestic porcine brain. To display our methodology, we imaged the putative cargo receptors Basigin, transferrin receptor and sortilin [11, 20, 28]. Using ExM combined with established capillary isolation techniques, we find that our approach enables hitherto unprecedented nanoscale analysis details for cargo receptor localization in ex vivo brain microcapillaries.

## Materials and methods

All plastic ware was obtained from Corning or Greiner BIO-ONE unless otherwise stated.

### Antibodies

**Table 1:** Study antibodies with functionality and origin

Primary antibody	Antibody usage	Dilution	Species / clonality	Source
Anti-Sortilin	Immunocytochemistry Western blotting	20 µg/ml 1 µg/ml	Mouse monoclonal	In-house developed
Anti-TfR	Immunocytochemistry Western blotting	10 µg/ml 0.5 µg/ml	Mouse monoclonal	ThermoFisher 13-6800 / 13-6890
Anti-Basigin	Immunocytochemistry Western blotting	20 µg/ml 1 µg/ml	Humanized Mouse monoclonal	In-house developed
Anti-collagen4	Immunocytochemistry	8 µg/ml	Goat polyclonal	Novusbio NBP1-26549
Anti-Mouse Alexa488	Immunocytochemistry	20 µg/ml	Donkey polyclonal	Invitrogen A21202
Anti-Human Alexa488	Immunocytochemistry	20 µg/ml	Donkey polyclonal	Biotium 13C0308
Anti-Goat StarRed (647)	Immunocytochemistry	10 µg/ml	Donkey polyclonal	Abberior STRED-1055
Anti-Beta actin	Western blotting	1:10.000	Mouse monoclonal	Sigma A5441
Anti-mouse HRP	Western blotting	1:2.000	Horse polyclonal	Cell Signaling 7076s
Anti-Human HRP	Western blotting	1:2.000	Goat polyclonal	Millipore AP112P

## Purification of porcine brain capillaries for expansion microscopy

Porcine brains were obtained as byproducts of the Danish food industry. Ten brains from 5-6-months-old domestic pigs were collected fresh from a nearby slaughterhouse and transported on ice to the laboratory. The brains were placed in a sterile flow bench and washed with 1 L PBS in a beaker on ice. Capillaries were isolated from the brains as described before [19, 22] using 140 μm and 41 μm filter-mesh (Sigma, Cat. no.: NY4H04700 and NY4104700). The filter-separated capillaries were collected in ice-cold PBS from filters and washed two times by centrifugation at 4 °C, 250 x g for 5 min in DMEM/F12 with 5% FBS and penicillin (100 U/mL), streptomycin (100 µg/mL). Capillaries were freeze stored (at -80 °C or in liquid nitrogen) in freezing medium containing DMEM/F12 with 5% FBS and penicillin (100 U/mL), streptomycin (100 µg/mL) with 10% DMSO. Capillaries from one brain were stored per 1 mL cryovial.

## Isolation of primary porcine and rat BECs for lysates

A non-contact Transwell co-culture was set up with astrocytes purified from 1 to 2 days old Sprague–Dawley rats seeded the in bottom chamber and porcine BECs (pBECs) seeded in the top chamber purified from 5-6-months-old porcine brain capillaries as previously described [19]. Following primary cell purifications, astrocytes and pBECs were cultured until the barrier reached a transendothelial electrical resistance (TEER) value > 1000 Ω cm^2^, after which pBECs were lysed and used for Western blotting. TEER values were measured using an EndOhm-12 measurement device (World Precision Instruments) as described before [20]. Capillaries from rat were isolated from 3-weeks rat as previously described [25].

## Western blotting

Cells or isolated capillaries were lysed in lysis buffer containing 1% Triton X-100, 150 mM NaCl, 2 mM MgCl_2_, 2 mM CaCl_2_, and complete mini protease inhibitor cocktail (Roche) for 30 min on ice with occasional vortexing. Lysates were spun down and supernatant lysates were collected. Lysates were added 20 mM DTT and NuPAGE LDS sample buffer (Invitrogen, NP0007). The mixture was heated to 95 °C for 5 min. Samples were loaded into 4–12% bis–tris gels next to SeeBlue™ Plus2 prestained protein standard as a protein marker in a running buffer prepared from 20x NuPAGE MES buffer, ddH_2_O and NuPAGE antioxidant. Samples were run for 45 min at 140 V. Gels were then transferred for blotting using a Novex iBlot dry blotting system (Invitrogen, cat# IB24001) and blocked in Tris-buffered saline (TBS) with 0.1% Tween-20 and 5% milk. The blots were incubated with primary antibodies at 4 °C overnight and washed in 0.1% Tween-20 in PBS, followed by incubation with secondary antibodies for 1 h at room temperature (RT). After washing in 0.1% Tween-20 in PBS, the blots were developed using ECL substrate (Pierce, 32106) and detected using an iBright 1500 (Invitrogen) chemiluminescence imager.

## Immunofluorescence staining of samples for expansion and confocal microscopy

Samples were fixed with cytoskeleton fixation buffer containing 10 mM MES, 3 mM MgCl_2_, 138 mM KCl, 2 mM EGTA, 0.32 M sucrose and 4% PFA for 15 min at RT. Cells were stained using conventional methodology, including 10 min of 0.2% Triton X-100 in PBS for permeabilization and 30 min of 2% BSA in PBS for blocking. Primary antibodies were diluted as listed in Table 1 (these dilutions were 5 times more concentrated than for standard immunofluorescent staining) in blocking solution and added the samples (1 h at RT for filter-seeded cells and overnight at 4 °C under rotation for capillaries). Samples were then washed and incubated with secondary antibodies diluted as described in Table 1 for (30 min for filter seeded cells and two hours at RT under rotation for capillaries). Samples were then washed in PBS before embedding with expansion gel.

### Sample gelation and expansion of isolated capillaries in solution

**Fig. 1:**
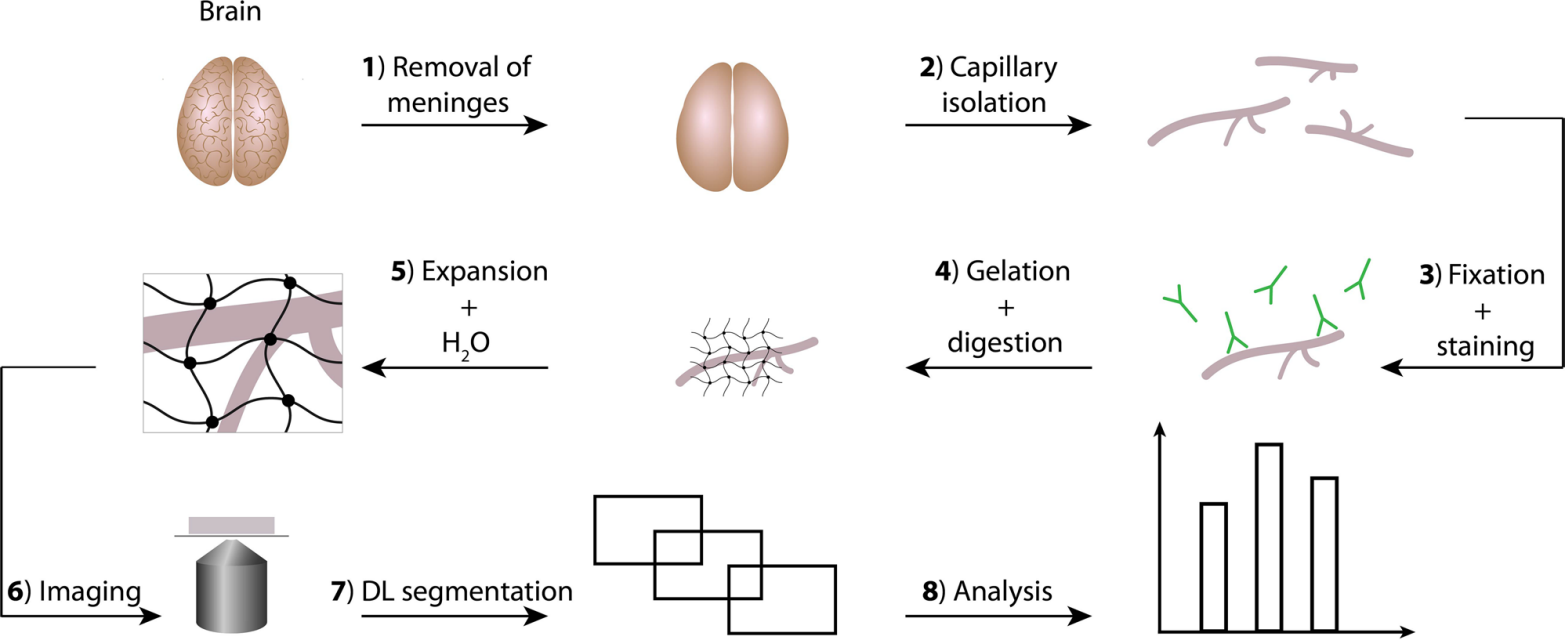
Technical overview of mapping procedure in brain capillaries. **1**–**2**) Meninges of porcine brains are removed and discarded before the grey cortex area containing penetrating vessels and capillaries are scraped of and isolated. Isolated capillaries are frozen. **3**) Frozen capillary fraction is thawed, fixed and immunostained for relevant markers and receptors of interest. **4**) A polyacrylamide gel is casted around the immunostained capillaries and when polymerized, the gelled capillaries are homogenized by proteinase K digestion to ensure isotropic expansion. **5**) Homogenized and gelled capillaries are placed in water and osmotic forces exchanges salts of the gel with water, which makes the acrylamide fold out and expand 5x in three dimensions. **6**) The 5x expanded gel is cut out to fit in an imaging chamber and imaged using a confocal spinning disc microscope with a 60x water objective. **7**) Images are generated in z-stacks and representative images are picked for deep learning (DL) trainings. DL is performed on selected images until marker and receptor signal can be covered in a segment and reported as spots. **8**) The trained spot segmenter is used to analyze all data and spot distances between receptors and marker are reported

As previously described [20], the expansion procedure consisted of an anchoring step with a cross-linker, a gelation step to embed proteins in a swell-able acrylamide gel via a cross-linker and an expansion step where washing with removed salt bridges between acrylamide polymers, gradually releasing and stretching the elastic acrylamide polymers (Fig. 1). Fixed and stained capillaries were pelleted following the last wash of the immunofluorescent staining and all liquid was removed before adding gelation solution. The cells were added an ice-cold droplet (40 µL) of 5x gelation solution (150 mM NaCl, 13.2% (w/w) acrylamide, 0.035% (w/w) N, N’-methylenebisacrylamide, 12.5% (w/w) sodium acrylate in PBS) with 0.2% Methacrolein added as cross-linker and left to incubate for 30 min. Then 0.01 wt% 4HT (4-Hydroxy-TEMPO), 0.2 wt% tetra-methyl-ethylene-diamide and 0.2 wt% ammonium persulfate were added to the droplet and a 13 mm round coverslip was placed on top. The gelation proceeded for 60–90 min under humidified conditions at RT until the gel had polymerized around the sample. The gel and cover glass were removed from the parafilm, placed in digestion buffer 0.5% Triton X-100, 0.8 M guanidine HCl in TAE buffer (40 nM Tris, 20 nM acetic acid), 1 mM EDTA with freshly added Proteinase K (0.8 units/mL), and incubated for 30 min in 37 °C at 200 RPM shaking. Subsequently, sample gels were placed in PBS at 4 °C overnight. The following day the gel was placed in distilled water on a rotating table and was exchanged three times at 30 min intervals. During the last water wash, 1:20.000 Hoechst 32,528 (Invitrogen H3569) was included for nuclei staining. The expanded specimens were cut to fit into a circular 35 mm Chamlide chamber (Live Cell Instruments, Cat. No.: CM-B-40) placed on a 25 mm coverslip fitting the bottom of the magnetic chamber.

## Image and distance analysis

Confocal images were generated from mounted capillaries or gel-expanded capillaries captured by an Olympus IX-83 fluorescence microscope with a Yokogawa CSU-X1 confocal spinning unit and a Hamamatsu Orca Fusion BT C15440 camera, Olympus UPLSAPO W, 60X/1.20 NA water objective lens (pixel size x: 108 nm y: 108 nm and z: 310 nm), using Olympus CellSens software (Olympus). For each channel, the laser power was adjusted and applied independently. Images were processed manually for presentation using IMARIS software v8.2 (Bitplane). Files were converted and analyzed using the Arivis AI module with deep learning segmentation (Arivis Vision4D vs. 4.1.1, Carl Zeiss Microscopy Software Center Rostock GmbH). Images of the gelled and expanded capillaries had a low signal-to-noise ratio due to the dilution of signal from 5x expansion in three dimensions resulting in 125 times signal dilution. The deep learning tool was used for segmentation training on selected images setting the 488 receptor and 647 collagen IV signals as 2 separate channels. Relevant pixel signals were chosen individually for the two channels and background was marked. The machine was trained in twelve cycles until relevant pixels were included and background was left out from the spot segmentation. A pipeline was generated using this segmentation setup reporting distances between 488 receptor and 647 collagen IV signals. A total of 8.400 confocal images from 60 z-stacks were segmented for analysis.

## Statistics

Statistical tests were performed using Prism (v.10) (GraphPad Software). All data sets are based on three or more independent experiments. Bar plots present mean values with standard deviation error bars. Different means were analyzed using the tests indicated in the figure legends with *p < 0.05, **p < 0.01, ***p < 0.001, ****p < 0.0001 and nonsignificant (ns).

## Results

Brain capillary vessels from domestic porcine brains were isolated according to the previously published protocol [19, 22], with the only change that the trypsin and collagenase digestion step prior to freezing of isolated capillaries was omitted. This deviation ensured conservation of receptor epitopes for immunostaining thereby retaining signal, which due to its 125 times dilution post 5x specimen expansion (three dimensions times 5x) by the expansion procedure otherwise would be insignificant. Frozen capillaries were thawed directly in fixative containing glucose and 4% PFA. Following a standard immunostaining procedure using 5x additionally added antibodies, capillaries were fixed between glass with mounting medium (Fig. 1, step 1–3).

### Crude capillary typing for imaging

**Fig. 2:**
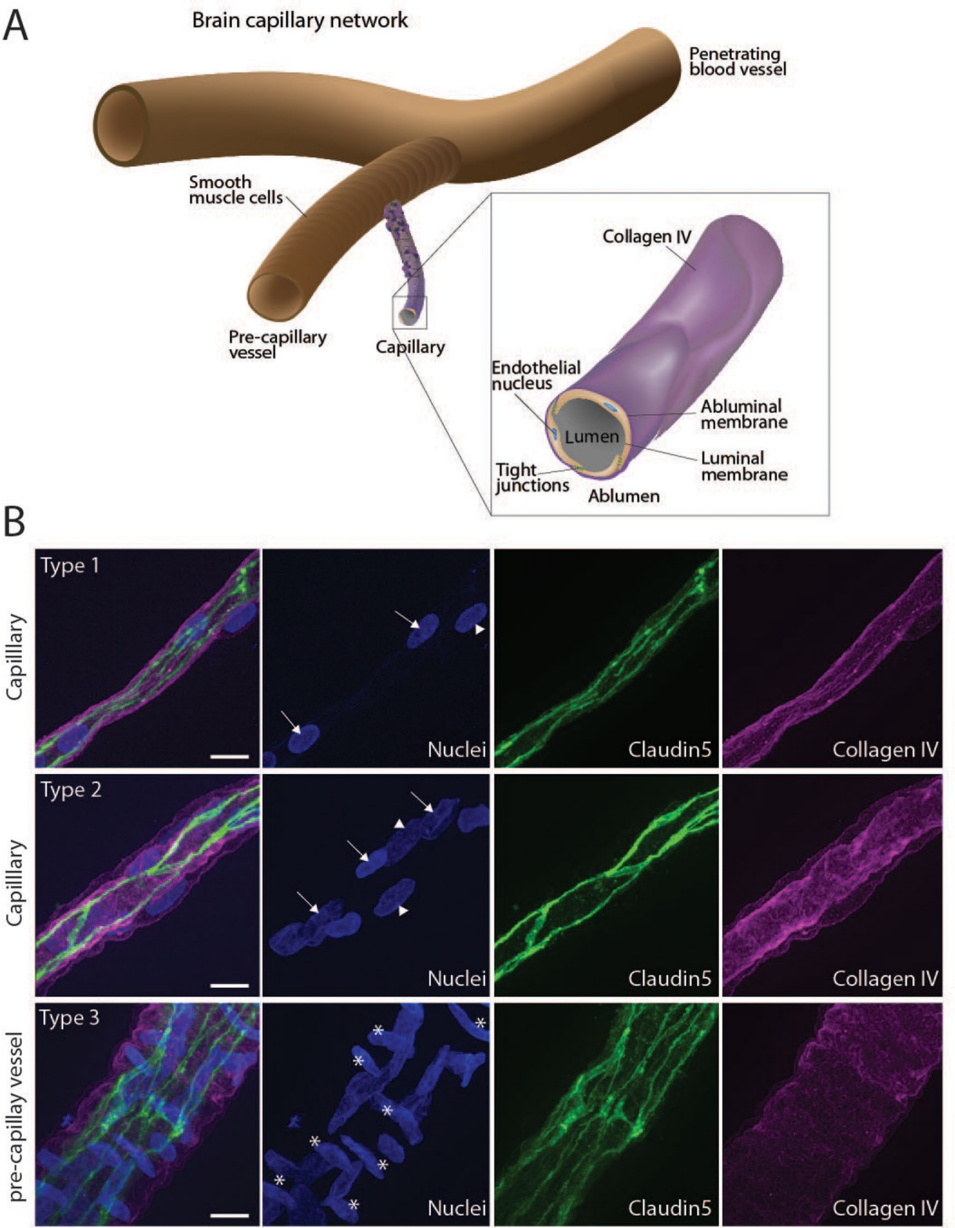
Typing of capillaries for expansion microscopy using nuclei density and orientation. **A** Overview illustration of brain capillary network isolated from porcine brain show penetrating blood vessel branching into precapillary vessels, both ensheathed by smooth muscle cells. Capillaries of interest branch from precapillary vessels and are not ensheathed by smooth muscle cells and hold only decreasing amounts of pericytes. **B** Representative micrographs of selected capillaries stained for the endothelial marker claudin5 and the abluminal surface marker collagen IV. Nuclei density and orientation characterize cellular populations with arrows marking endothelial cell nuclei, arrowheads suggesting pericyte nuclei and asterisks suggesting smooth muscle cell nuclei. Scale bars = 10 μm. See also additional figure S1

Mixed pre-capillary and capillary vessels were observed, and crude typing of capillaries was performed based on nuclei density and positioning (Fig. 2B nuclei stain for crude capillary typing; Fig. S1 examples of stained and mixed capillaries). Microcapillaries, i.e. type 1 capillaries, were defined by cell nuclei forming a discontinuous line (arrows) harboring only few peripheral nuclei (arrowhead). Type 2 capillaries were also characterized by nuclei forming a discontinuous line but with several nuclei surrounding those (arrowheads). Type 3 capillaries were characterized by their resemblance to previously described pre-capillary vessels harboring cell nuclei surrounding a longitudinal luminal nuclei population facing in perpendicular direction (asterisks), as described for smooth muscle cells on penetrating arterioles [8, 9]. The crude typing of capillaries based on nuclei density and orientation was necessary to generate a clear signature for identifying microcapillaries in the mixed capillaries embedded in the expanded gel. The gelled and expanded gel was much larger than the imaging area; therefore, we introduced the capillary typing based on nuclei density and orientation to aid the later identification of type 1 capillaries of interest in the gelled and expanded samples.

### Verification of capillary integrity

The integrity of the processed and stained capillaries was tested using the endothelial tight junctional marker Claudin5 and the capillary marker collagen IV (Fig. 2A for overview).

Imaging of capillaries of the respective types showed intact staining patterns both for Claudin5 and collagen (Fig. 2B).

### Capillary expression of putative cargo receptors

**Fig. 3:**
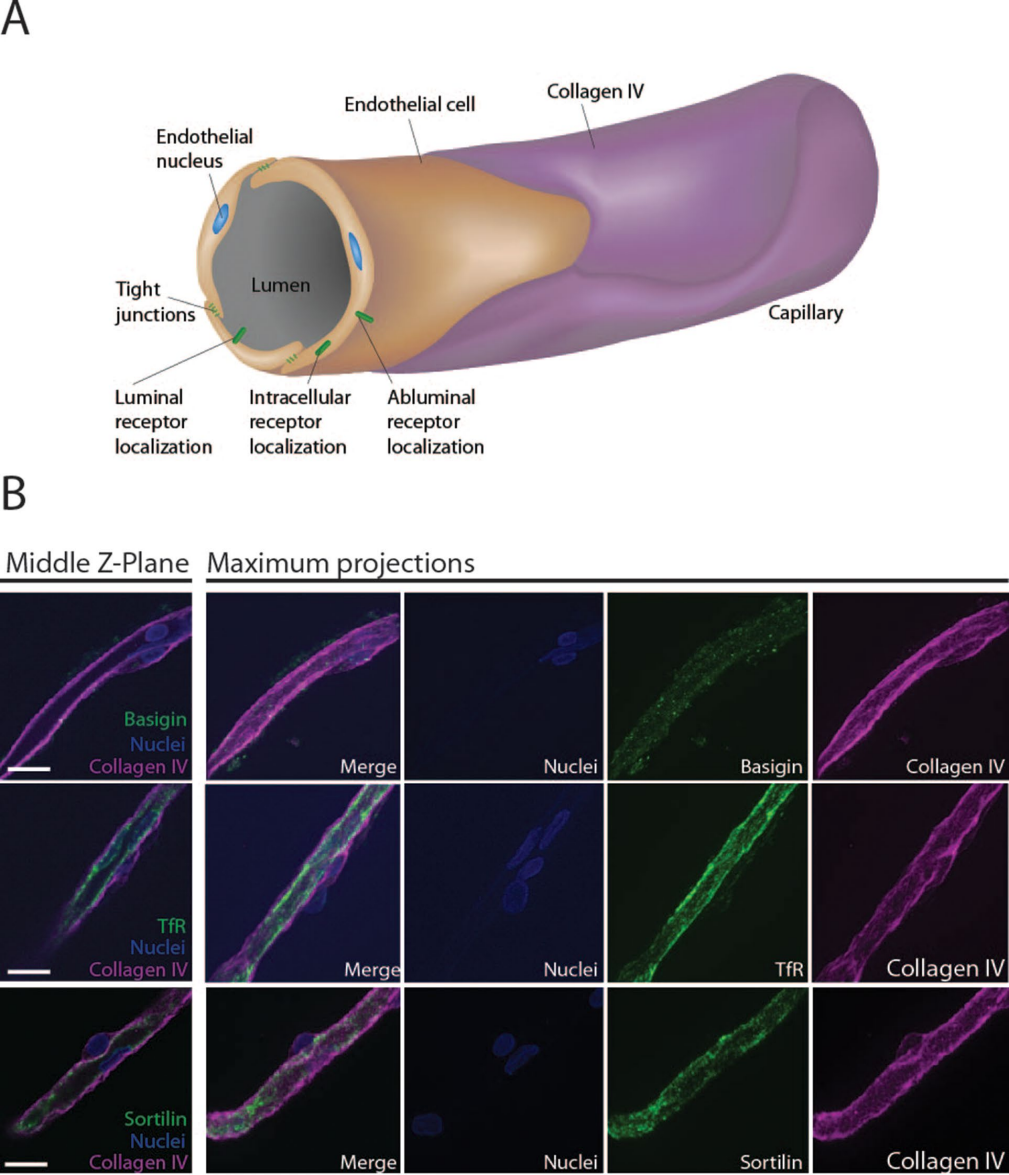
Expression of basigin, TfR and sortilin in type 1 capillaries. Overview illustration of a type 1 capillary coated with the abluminal marker collagen IV In type 1 capillaries, a thin monolayer of endothelial cells are interconnected by overlapping processes and tight junction proteins thereby forming the capillary structure. Endothelial cells express receptors, which localize on luminal membranes, intracellularly and on the abluminal membrane surface. Representative micrographs of single micrographs from z-stacks and maximum projected z-stacks of type 1 capillaries. Scale bars = 10 μm. See also additional figure S2

Various receptors are expressed in brain endothelial cells, which may be relevant as cargo receptors for delivering therapeutics. Here we tested the expression of three prominent receptors to further delineate the receptor localization in an ex vivo model. We chose transferrin receptor (TfR), sortilin and basigin, which have been found to be expressed in BECs [11, 20, 25, 28]. We used previously characterized antibodies [3, 11] targeting these receptors and performed immunocytochemistry and Western blotting to determine the expression of receptors in isolated porcine brain capillaries (Fig. 3 and Fig. S2).

The 3D volume and molecular locations in collagen IV seeded in vitro cell cultures are simple sources to obtain high quality micrographs and to generate super-resolution data for molecular analysis of receptor localizations [20]. To advance the usage of this setup ex vivo we considered that in capillaries, collagen IV covers the abluminal surface of capillaries whereas the glycan saturated glycocalyx covers the luminal surface of capillaries devoid of collagen IV [26]. This makes collagen IV an excellent marker for the surface of the abluminal membrane of the here chosen type 1 capillaries, where only BECs are forming the capillaries. Basigin showed low expression in the brain endothelial cell layer with signal appearing overlapping with the collagen IV stain (Fig. 3 middle Z-plane and Fig. S2). Both TfR and the newly characterized putative cargo receptor sortilin, showed high expression in type 1 capillaries with expression dispersed in the endothelial cell layer (Fig. 3 middle Z-planes and Fig. S2).

### Image spot segmentation using deep learning

**Fig. 4:**
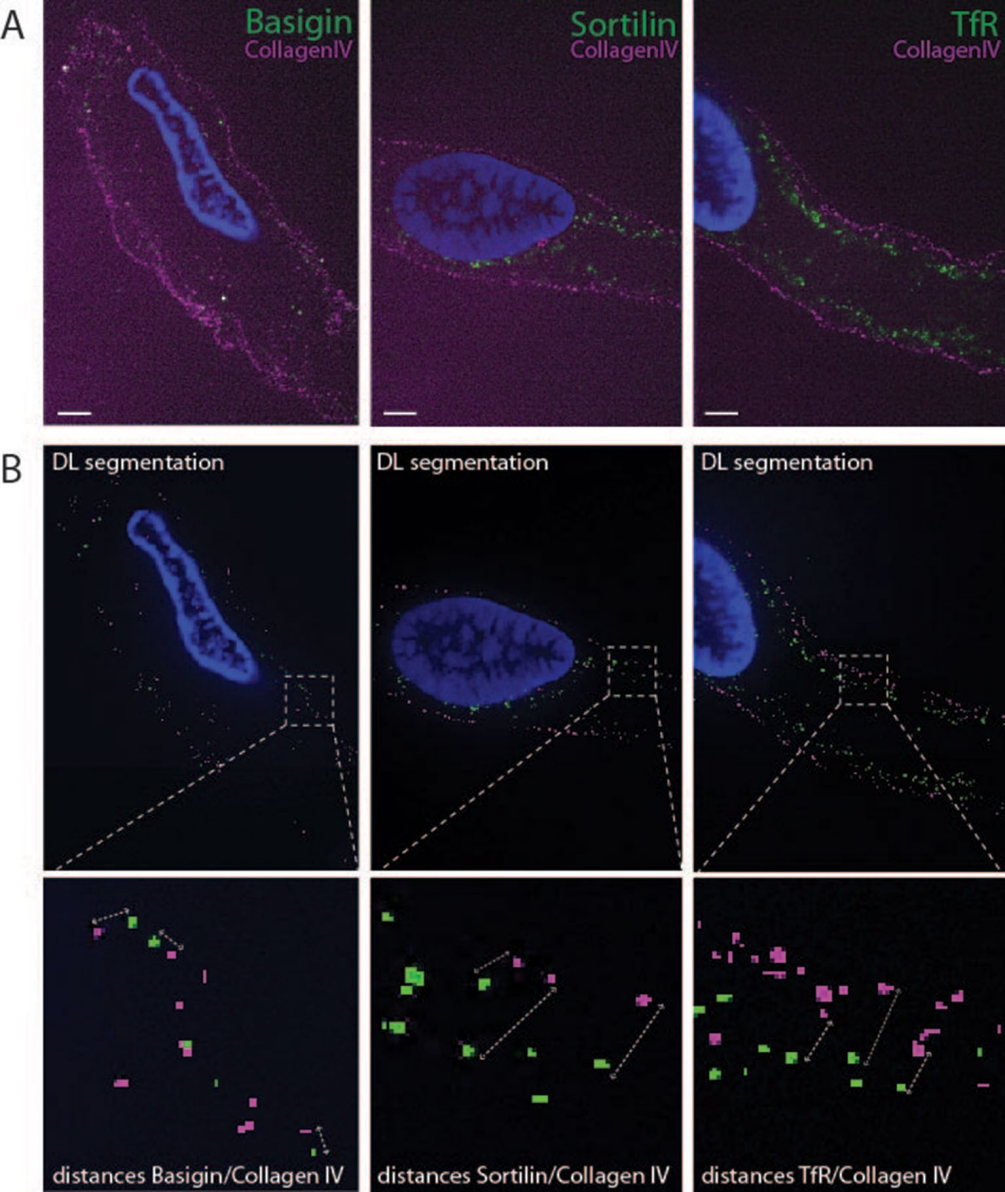
Analysis of receptor localization in expanded capillaries. **A** Representative confocal z-plane of middle sections of 5x expanded capillaries stained for nuclei (blue), the indicated receptor (green) and collagen IV (magenta). Scale bars = 2 μm. **B** Representative overlay of segmented spots from AI analysis. Receptors green spots) and collagen IV (magenta spots). Magnified insets show the pipeline measurements of distances between receptors and nearest collagen IV spot. See additional figure S4 for micrograph training example

After confirmation of receptor expression and specificity of chosen analytic antibodies, we proceeded to test if expansion microscopy could be used to obtain super-resolution of receptors in the BEC layer of type 1 capillaries. Expansion microscopy is a fairly simple procedure but there are limitations when applying the technique to tissue samples such as capillary tissue [23]. We combined our previous protocol [20] with recent advances from the MAGNIFY protocol [14] by including methacrolein into the polyacrylamide gel solution to by-pass the anchoring step. This approach and longer digestion time enabled us to obtain 5x expanded capillaries with sufficient signal for image analysis (see Fig. 4B). Two circumstances affect signal retention in expanded samples: (1) the expansion ratio where 5x expansion in three dimensions gives 125x signal dilution and (2) the loss of signal during homogenization of the gelled sample to enable isotropic expansion. Here we successfully obtained a signal-to-noise ratio suitable for spot segmentation using 30 min homogenation by proteinase K digestion. Due to the low signal to noise ratio from the gelled sample, we were unable to use conventional imaging analysis software. Therefore, we took advantage of newly developed artificial intelligence (AI) tools (Arivis). Training of the machine was performed in 12 rounds using the AI module of the software package (Fig. 4 middle panels). In short, areas in selected micrographs were used, and herein, two classes of pixels were segmented by hand and a background mask was included to show the machine what was considered signal and noise. This was done in selected areas of micrographs in five of the many obtained micrographs containing receptor channel (Class 1 pixels) and abluminal membrane marked by collagen IV (Class 2 pixels), limiting the hands-on time (Fig. S4). After twelve trainings, the resulting segmentation setup enabled a satisfying coverage of signal. The resulting segmenting setup was then used in a pipeline to employ on the entire data set. Here the receptors and collagen IV were segmented individually and the closest distance between the respective receptors and collagen IV was computed (Fig. 4B lower panels).

### Analysis of abluminal receptor localizations

**Fig. 5:**
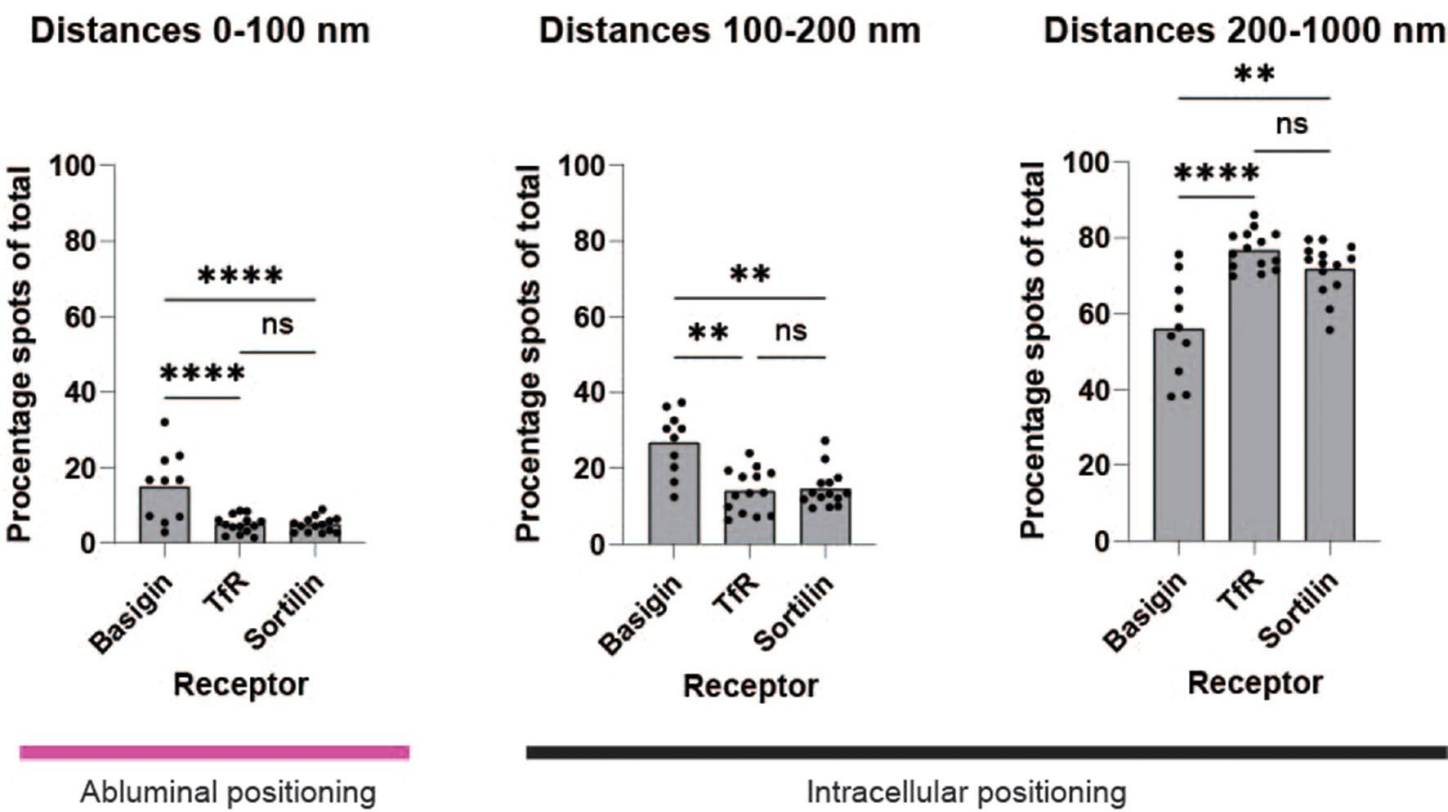
Nanoscale measurement of abluminal receptor localizations. Bar plots of measured distances between collagen IV and receptors. Data is collected for basigin (*n* = 10 capillaries), TfR (*n* = 14 capillaries) and sortilin (*n* = 14 capillaries) from three independent experiments. Distances ranging from 0–100 nm are considered abluminal receptor localization and 100–1000 nm are considered intracellular receptor localization. Statistical significance was tested by two-way ANOVA with Tukey´s multiple comparisons test. See also additional figure S5 for residual distances measured between 1–3 μm

To understand how the three chosen receptors distributed in the ex vivo BECs, we analyzed the distances between receptors and collagen IV. We divided the measured distances into what is considered abluminal localizations in 0–100 nm distances and intracellular localizations in 100–1000 nm distances (Fig. 5). Both 0–100 nm and 100–200 nm distance measurements showed presence of all three receptors, but for basigin a significantly higher number of receptors was found in the abluminal area located in 0–100 nm proximity to collagen and for the 100–200 nm intracellular localizations. This distribution was also reflected by a significantly lower presence of basigin in 200–1000 nm intracellular localizations. Remaining localizations at 1–3 μm distance from collagen IV showed no significant difference amongst the receptors (Fig. S5).

## Discussion

Brain capillary vessels from domestic porcine brains are a rich source for acquiring experimental in vivo tissue with close relationship to the human genome, anatomical size, immunology and therefore constitutes an important tool for translational medical research [16]. Refinement of protocols for isolation of the large amount of available material have resulted in high quality isolated primary BECs as well as brain capillaries to study BECs in their barrier environment [19, 22]. Here, we refined our previous protocol to improve the usage of capillaries for imaging after isolation. The previously used digestion step [19] was omitted and an extra filtration step with a smaller pore size (41 μm) was included to increase the amount of isolated thin microcapillaries running through the initial 100 μm filtering step. Verification of the quality of the obtained capillaries using collagen IV and claudin5 immunostaining showed high numbers of intact capillaries with varying complexity (Fig. 2). Typing of these and testing for receptor expression of interest, showed presence of basigin, TfR and sortilin in type 1 capillaries in focus (Figs. 2 and 3), where basigin was expressed the least. Usage of collagen IV as a BEC abluminal marker was relevant for type 1 capillaries but not for type 2 and 3 capillaries. The multiple cell layers in type 2 and 3 capillaries intertwined with additional layers of collagen IV from pericytes and mural cells disrupted precise localization of the endothelial abluminal membrane thereby complicating the usage of collagen IV as a purely endothelial abluminal marker. However, as focus was kept on type 1 capillaries, the usage of collagen IV as an abluminal membrane marker proved excellent for further analysis of abluminal receptor localization in ex vivo BECs.

In theory, microcapillaries hold the best promise for delivering of drugs deep into the neuronal cell layer due to their short distance to virtually the entire population of brain neurons [5, 18]. History shows that the best strategy for drug delivery to the brain is via a BBB drug delivery strategy using a cargo receptor like TfR [21]. With recent findings that the brain vascular endothelium exerts large spatial transcriptomic heterogeneity [7, 12], it becomes warranted to investigate both spatial expression as well as intracellular localization of putative drug carriers such as TfR. This will help to evaluate their applicability to transcytose designed drugs through the brain endothelium to reach their target neurons.

Current conventional microscopy techniques allow imaging of tissues at a cellular resolution. This resolution limits the investigation of possible intracellular endothelial drug transport pathways due to the slim nature of BECs. However, to obtain more knowledge of the transport of intracellular endothelial cargo receptors, imaging the brain vascular endothelium at nanoscale resolution is necessary. Super resolution imaging has been developed using expansion microscopy on whole brains [6] but the technique is time consuming, expensive and generates data with extreme complexity. As an attempt of simplification, we aimed to obtain images of ex vivo brain capillaries at nanoscale resolution. We chose the three putative cargo receptors basigin, TfR and sortilin exampling both low (basigin) and high (TfR and Sortilin) receptor expression in the brain vascular endothelium of pig (Fig. 3 and S2). The technique outlined in Fig. 1 can be performed on any species of choice.

To evaluate the effects on capillary tropism of using the expansion method we compared imaged capillaries before and after expansion (Fig. S3A). The examples of immunostained capillaries we visually inspected before and after expansion showed little distortions, suggesting good isotropic expansion. We performed visual inspection of capillary nuclei size to find and choose uniform expanded capillaries. We only choose uniform expanded capillaries and disregarded capillaries, which did not have uniform nuclei sizes (these instances did occur in the gel and should be disregarded).

To further evaluate the method we used a GFAP as astrocyte marker next to a VE-Cadherin as BEC marker stain to display the method. This labelling enabled us to show leftovers of astrocyte endfeets above the BEC cell layer, which is not possible with conventional microscopy (Fig. S3B). This highlights the superior resolution obtained when imaging capillaries using expansion microscopy. This also opens the possibility of planning future studies using GFAP antibody together with molecules of interest to obtain new knowledge of the interactions around astrocyte endfeets in health and disease.

The applicability using this protocol on other species was tested using freshly isolated rat capillaries (Fig. S3C). The method is expected to work on mice and other species as well but it should be noted that the staining of soluble material includes several washes and the subsequent expansion will dilute the density of the capillaries in the gel. Therefore, there is a practical need of using several brains if working with mice and other small species to ensure enough material. Although the optimization of specific antibodies are comprehensive for different species, the method is expected to be applicable to study many different markers for quantitative localization studies in capillaries.

A major limitation of expansion microscopy is the dilution of signal after expanding the sample and we found this to be an issue for our setup. Especially when imaging of basigin we found a very low signal-to-noise ratio in the images of the expanded microcapillaries. We were unable to segment using conventional spot segmentation algorithms and therefore applied deep learning (DL) spot segmentation to pick out the basigin signal from background noise of the image. This approach proved very efficient for filtering (Fig. 4) and helped to automate the data curation of 8,400 micrographs used for this study. DL and AI are becoming powerful tools in science and will be used on a routine basis in image analysis [17].

We recently reported in vitro data, which suggests exclusively luminally localized receptors to be inefficient for therapeutic antibody transcytosis [11]. TfR is found functional for in vivo transcytosis of cargo antibodies [27] and our previous in vitro ExM localization analysis has shown abluminal localization of TfR [20]. Likewise, ex vivo, we find app. 8% of TfRs to be localized abluminally (Fig. 5). Interestingly, sortilin displays a similar abluminal distribution as TfR ex vivo (Fig. 5) but with no apparent transcytosis activity in vitro [11]. Basigin is another putative cargo receptor, but its cellular localization has not yet been determined at nanoscale resolution. Here, we analyzed its localization ex vivo and found it to be significantly more abluminally localized compared to the two sorting receptors TfR and Sortilin with 32% of the receptor localized at the abluminal membranes marked by collagen IV (Fig. 5).

Here, we report a low-cost method to analyze the nanoscale localization of putative cargo receptors ex vivo. We focused on the abluminal localization of receptors in relation to collagen IV, but the method allows for analysis of localizations in relation to many other markers of interest. Especially the intracellular localization of the sorting receptors sortilin and TfR seem warranted, as these two receptors show opposing transcytosis capacity in spite of their similar localization [11]. Investigation of their respective intracellular transport paths using nanoscale analysis will likely aid to the understanding of drug transport paths for future design of targeted drug delivery at the BBB.

## Electronic supplementary material

Below is the link to the electronic supplementary material.

Supplementary Material 1

## Data Availability

No datasets were generated or analysed during the current study.
