# Supplementary material for: Ex vivo nanoscale abluminal mapping of putative cargo receptors at the blood-brain barrier of expanded brain capillaries
Source: Fluids Barriers CNS. 2024 Oct 14;21:80. doi: 10.1186/s12987-024-00585-x (PMC11475543; doi:10.1186/s12987-024-00585-x)
Supplement: Supplementary file 1 — Supplementary Material 1 [file 12987_2024_585_MOESM1_ESM.pdf]

Supplementary information

Ex vivo nanoscale abluminal mapping of putative cargo receptors at the blood-brain barrier of expanded brain capillaries

Mikkel Roland Holst[1], Mette Richner[1], Pernille Olsgaard Arenshøj[1],

Parvez Alam[1, 2] Kathrine Hyldig[1, 3] and Morten Schallburg Nielsen[1]*

This document includes:

Supplementary figures with figure legends

Supplementary figures

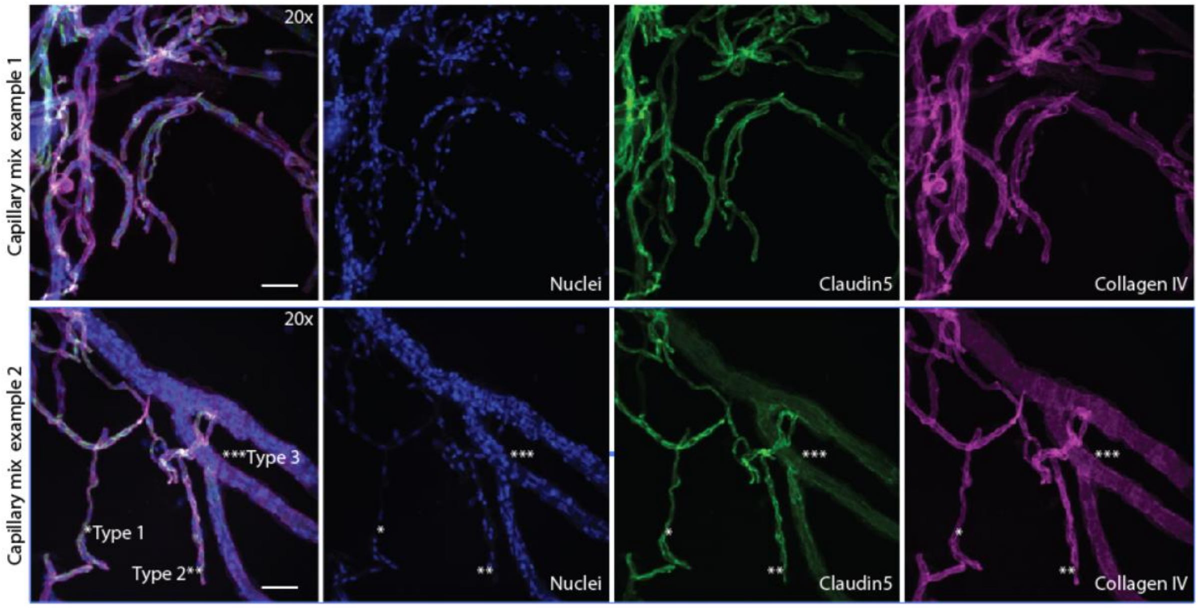

Figure S1. Example of immuno-stained capillary mix. Representative maximum projected z-stacks of micrographs generated using spinning disk microscopy with an 20x objective. The mixed isolated and immune-stained capillaries show penetrating, pre-capillary vessels and capillaries. In capillary mix 2, type 1-3 capillaries are marked with asterisks based on nuclei density and orientation. Scale bars = 50 µm.

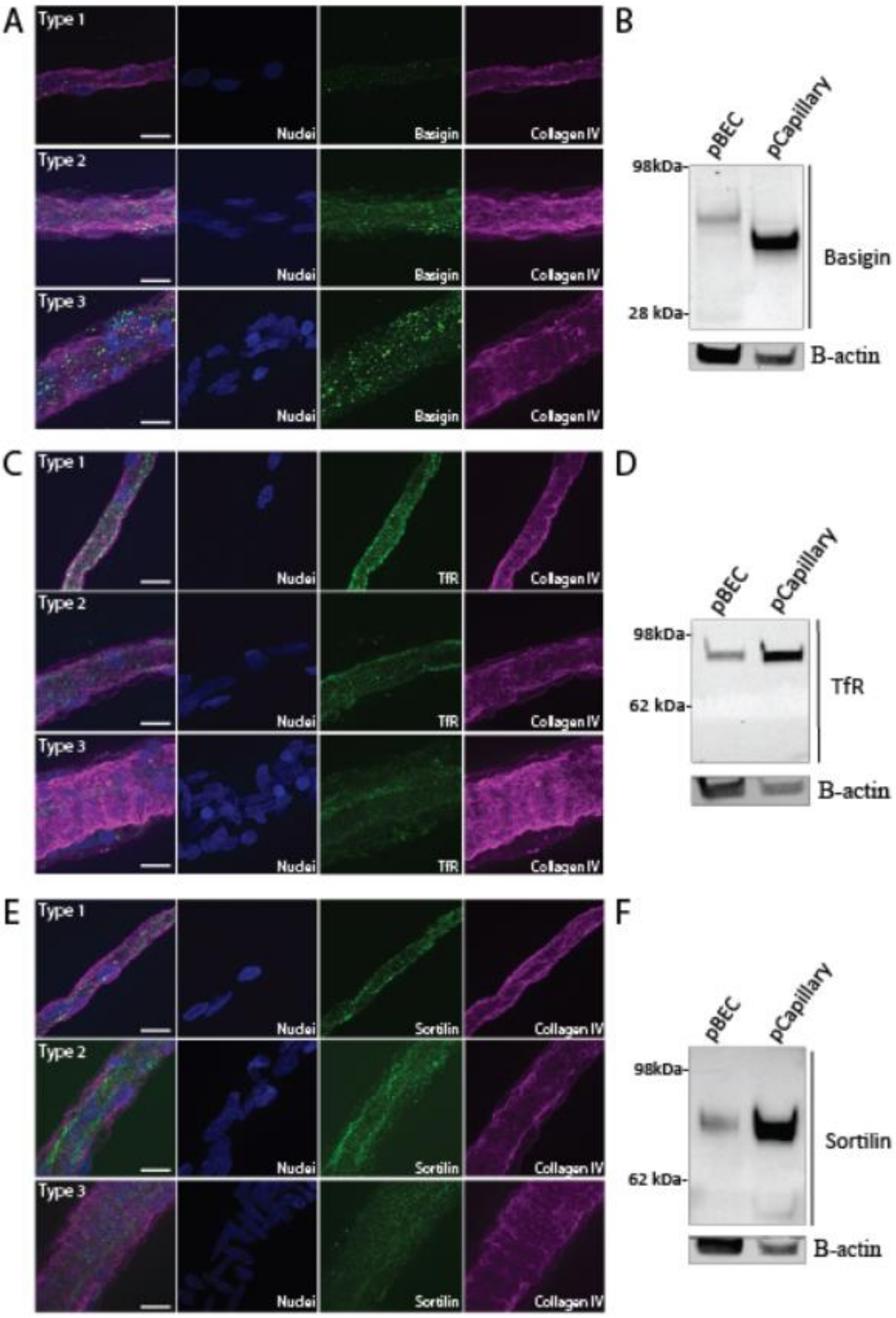

Figure S2. Receptor expression in relation to endothelial cells. A Representative maximum projected z-stack micrographs of imaged type 1-3 capillaries. Basigin is expressed throughout capillary cells with low expression in endothelial cells. Both TfR and sortilin are expressed throughout capillary cells with high expression in endothelial cells. Scale bars = 10 µm. **B** Representative Western blotting analysis of lysates from *in vitro* isolated porcine brain endothelial cells (pBEC) and isolated porcine whole-capillary mix (pCapillary). Western blots show abundant expression of the chosen receptors in capillaries and decreased expression when endothelial cells are isolated and cultured *in vitro*.

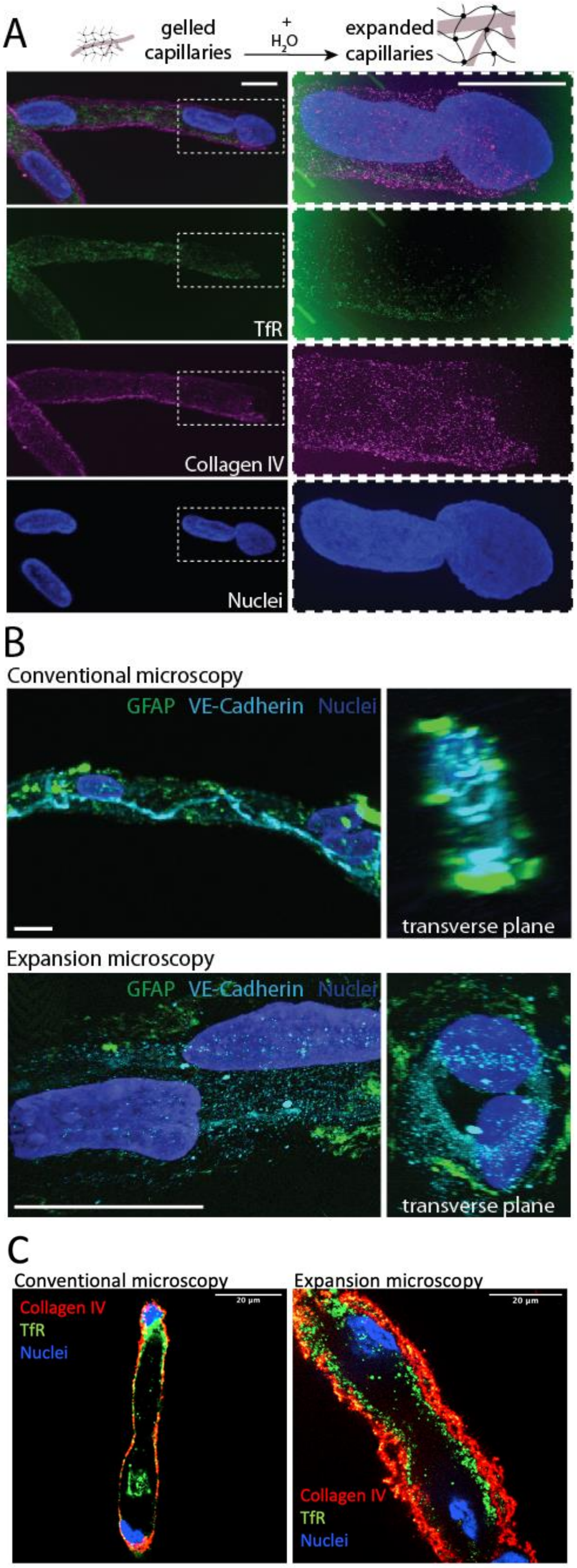
A
gelled capillaries
+ H2O
expanded capillaries
TfR
Collagen IV
Nuclei
B
Conventional microscopy
GFAP VE-Cadherin Nuclei
transverse plane
Expansion microscopy
GFAP VE-Cadherin Nuclei
transverse plane
C
Conventional microscopy
Collagen IV
TfR
Nuclei
20 μm
Expansion microscopy
20 μm
Collagen IV
TfR
Nuclei

Figure S3. Specification of isotrophic expansion and resolution increase. A Maximum projected micrographs of z-stacks. Immunostained and gelled porcine capillaries were first located in 3x5 mm gels and imaged. For gel and capillary expansion, the 3x5 gels were incubated with water for 4x 30 minutes adding the nuclei dye Hoechst to the last water incubation. Previous Imaged capillaries were relocated in the expanded gel and imaged again, as depicted. Scale bar = 10 µm. **B** Maximum projected micrographs of z-stacks. The astrocyte marker GFAP and endothelial marker VE-Cadherin was used to stain capillaries and illustrate the usage of expansion microscopy to increase image resolution to separate cell layers. The GFAP stain suggest the remaining presence of astrocyte endfeets after capillary isolation Scale bar = 10 µm. **C** Single plane of immunostained rat capillaries with collagenIV (red), TfR (green) and nuclei (blue). Expanded as porcine capillaries in A. Scale bar = 20 µm.

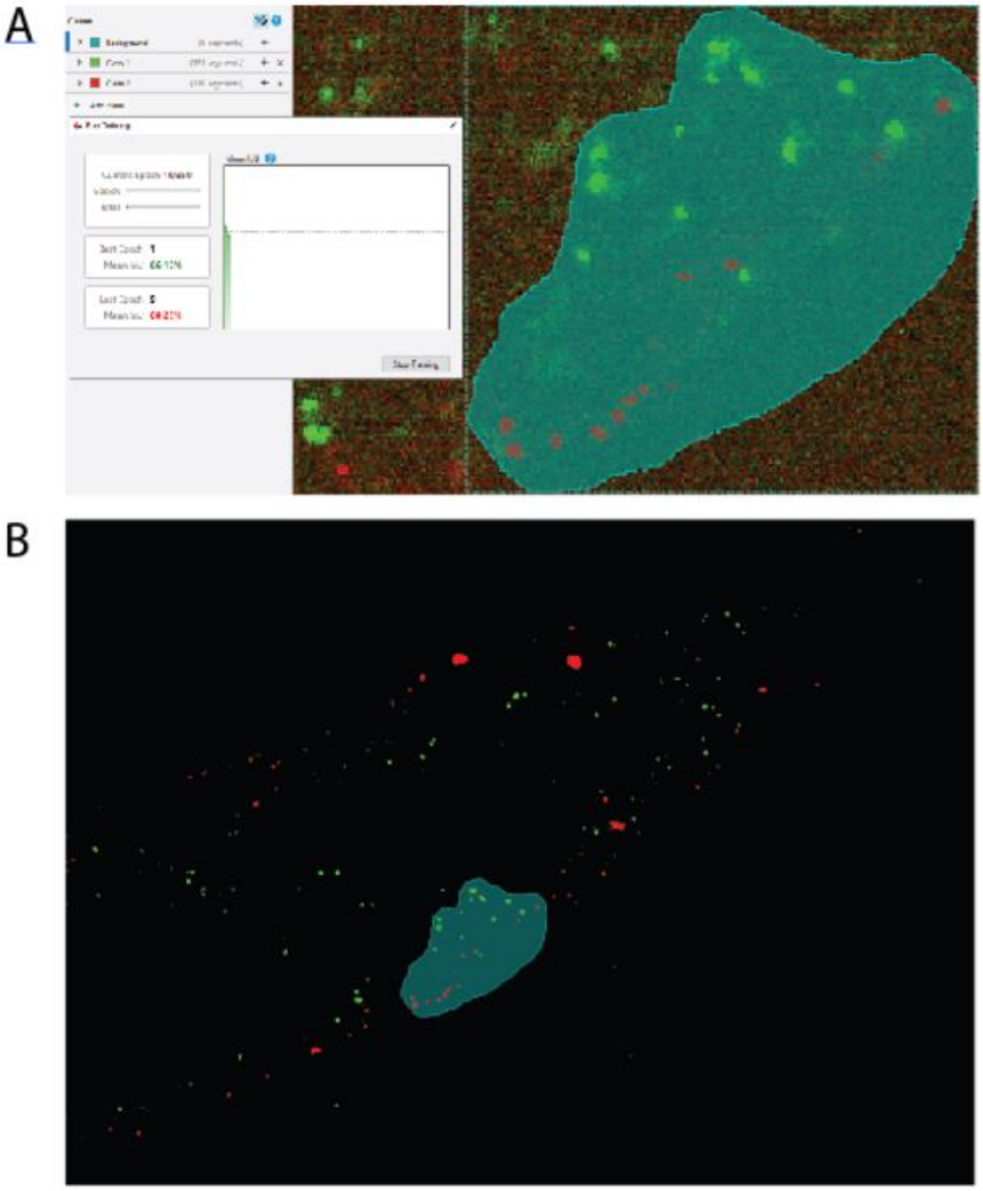

Figure S4. Deep learning training interface. A Screenshot of AI training module (Arivis). Class 1 pixels (receptor signal from the green 488 channel) and class 2 pixels (collagen IV signal from the red 647 channel) are manually drawn pixel by pixel and a background mask is drawn around the selected pixels to mark noise in the raw image. The training session is started and the deep learning runs (epoch is the measure of UNIX time). **B** The micrographs show the blowout of the entire micrographs with selected training area from **A.** After the training session has completed on the selected micrograph, the user is informed with an image of the newly segmented spots on a black background. Manual inspection is then performed to evaluate if the resulting DL algorithm segments the imaging data correctly or if additional training rounds are required.

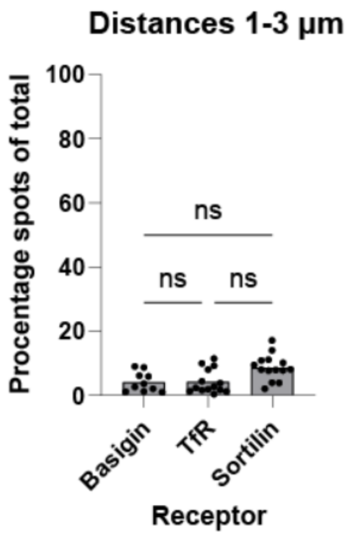

Figure S5. Residual distances. Bar plot shows the measured distances between collagen IV and receptors. Data is collected from basigin (n=10 capillaries), TfR (n=14 capillaries) and sortilin (n=14 capillaries) from three independent experiments. Statistical significance was tested by two-way ANOVA with Tukey´s multiple comparisons test.
